# Supplementary material for: Modeling glioblastoma heterogeneity as a dynamic network of cell states
Source: Mol Syst Biol. 2021 Sep 16;17(9):e10105. doi: 10.15252/msb.202010105 (PMC8444284; doi:10.15252/msb.202010105)
Supplement: Supplementary file 5 — Source Data for Figure 3 [file MSB-17-e10105-s001.zip › Figure3A_sourcedata/GSEA_3065/nosigdb_state3.GseaPreranked.1622718426028/RICHARDS_ZHONG_NATURE_2018_OPC_UPREG.html]

Details for gene set RICHARDS\_ZHONG\_NATURE\_2018\_OPC\_UPREG[GSEA]

|  || Dataset | state3 |
| Phenotype | NoPhenotypeAvailable |
| Upregulated in class | na\_pos |
| GeneSet | RICHARDS\_ZHONG\_NATURE\_2018\_OPC\_UPREG |
| Enrichment Score (ES) | 0.6235883 |
| Normalized Enrichment Score (NES) | 2.2809527 |
| Nominal p-value | 0.0 |
| FDR q-value | 0.0 |
| FWER p-Value | 0.0 |
Table: GSEA Results Summary

  

Fig 1: Enrichment plot: RICHARDS\_ZHONG\_NATURE\_2018\_OPC\_UPREG      
 Profile of the Running ES Score & Positions of GeneSet Members on the Rank Ordered List

  

| PROBE | GENE SYMBOL | GENE\_TITLE | RANK IN GENE LIST | RANK METRIC SCORE | RUNNING ES | CORE ENRICHMENT || 1 | BCAN |  |  | 0 | 0.883 | 0.0577 | Yes |
| 2 | SCRG1 |  |  | 3 | 0.645 | 0.0997 | Yes |
| 3 | B2M |  |  | 11 | 0.538 | 0.1340 | Yes |
| 4 | PTPRZ1 |  |  | 14 | 0.532 | 0.1686 | Yes |
| 5 | PLAT |  |  | 18 | 0.513 | 0.2018 | Yes |
| 6 | PTN |  |  | 22 | 0.493 | 0.2337 | Yes |
| 7 | CD63 |  |  | 26 | 0.480 | 0.2647 | Yes |
| 8 | CD9 |  |  | 32 | 0.473 | 0.2950 | Yes |
| 9 | ALCAM |  |  | 45 | 0.440 | 0.3224 | Yes |
| 10 | PON2 |  |  | 63 | 0.389 | 0.3459 | Yes |
| 11 | SOX6 |  |  | 88 | 0.358 | 0.3665 | Yes |
| 12 | GPM6B |  |  | 105 | 0.343 | 0.3871 | Yes |
| 13 | PDGFRA |  |  | 115 | 0.326 | 0.4074 | Yes |
| 14 | SEMA5A |  |  | 121 | 0.324 | 0.4280 | Yes |
| 15 | BCHE |  |  | 136 | 0.315 | 0.4469 | Yes |
| 16 | TTYH1 |  |  | 183 | 0.293 | 0.4608 | Yes |
| 17 | NOVA1 |  |  | 270 | 0.253 | 0.4674 | Yes |
| 18 | ITM2C |  |  | 273 | 0.252 | 0.4836 | Yes |
| 19 | EDNRB |  |  | 305 | 0.242 | 0.4958 | Yes |
| 20 | LSAMP |  |  | 307 | 0.241 | 0.5115 | Yes |
| 21 | SERPINE2 |  |  | 308 | 0.241 | 0.5272 | Yes |
| 22 | SPARC |  |  | 361 | 0.228 | 0.5361 | Yes |
| 23 | CADM2 |  |  | 416 | 0.215 | 0.5440 | Yes |
| 24 | ITGB8 |  |  | 463 | 0.206 | 0.5521 | Yes |
| 25 | LRRC4C |  |  | 509 | 0.197 | 0.5598 | Yes |
| 26 | EPB41L2 |  |  | 596 | 0.187 | 0.5621 | Yes |
| 27 | OLIG1 |  |  | 602 | 0.186 | 0.5737 | Yes |
| 28 | SCD5 |  |  | 619 | 0.184 | 0.5839 | Yes |
| 29 | LIMA1 |  |  | 639 | 0.180 | 0.5935 | Yes |
| 30 | SLC35F1 |  |  | 711 | 0.168 | 0.5963 | Yes |
| 31 | CSPG5 |  |  | 716 | 0.168 | 0.6068 | Yes |
| 32 | CST3 |  |  | 749 | 0.164 | 0.6139 | Yes |
| 33 | ANGPTL2 |  |  | 758 | 0.162 | 0.6236 | Yes |
| 34 | ETV1 |  |  | 963 | 0.141 | 0.6093 | No |
| 35 | APOD |  |  | 1113 | 0.129 | 0.6006 | No |
| 36 | KANK1 |  |  | 1398 | 0.108 | 0.5749 | No |
| 37 | SPRY4 |  |  | 1461 | 0.105 | 0.5746 | No |
| 38 | SPRY1 |  |  | 1471 | 0.105 | 0.5805 | No |
| 39 | TSPAN7 |  |  | 1738 | 0.089 | 0.5557 | No |
| 40 | OLIG2 |  |  | 1742 | 0.089 | 0.5611 | No |
| 41 | SPATA6 |  |  | 1796 | 0.087 | 0.5607 | No |
| 42 | MMP2 |  |  | 1898 | 0.081 | 0.5544 | No |
| 43 | CD82 |  |  | 1930 | 0.079 | 0.5560 | No |
| 44 | EPN2 |  |  | 2164 | 0.070 | 0.5337 | No |
| 45 | TRIB2 |  |  | 2296 | 0.065 | 0.5228 | No |
| 46 | RFTN2 |  |  | 2346 | 0.063 | 0.5213 | No |
| 47 | PDE4B |  |  | 2443 | 0.059 | 0.5141 | No |
| 48 | ARC |  |  | 2798 | 0.047 | 0.4764 | No |
| 49 | SOX8 |  |  | 3470 | 0.029 | 0.4010 | No |
| 50 | NXPH1 |  |  | 3773 | 0.021 | 0.3676 | No |
| 51 | LMF1 |  |  | 4056 | 0.014 | 0.3360 | No |
| 52 | ETV5 |  |  | 4087 | 0.013 | 0.3334 | No |
| 53 | RAMP1 |  |  | 4100 | 0.013 | 0.3328 | No |
| 54 | FIBIN |  |  | 4788 | -0.006 | 0.2540 | No |
| 55 | SIRT2 |  |  | 4877 | -0.008 | 0.2444 | No |
| 56 | CNTN1 |  |  | 5924 | -0.037 | 0.1263 | No |
| 57 | GATM |  |  | 6161 | -0.045 | 0.1021 | No |
| 58 | DUSP6 |  |  | 6502 | -0.056 | 0.0666 | No |
| 59 | ANXA5 |  |  | 6522 | -0.057 | 0.0681 | No |
| 60 | SEZ6L |  |  | 6920 | -0.077 | 0.0274 | No |
| 61 | GNG7 |  |  | 7158 | -0.091 | 0.0061 | No |
| 62 | PLEKHB1 |  |  | 7359 | -0.105 | -0.0101 | No |
| 63 | TSC22D4 |  |  | 7610 | -0.129 | -0.0305 | No |
| 64 | PSAT1 |  |  | 7634 | -0.131 | -0.0246 | No |
| 65 | ARL4A |  |  | 7829 | -0.155 | -0.0368 | No |
| 66 | CNP |  |  | 7852 | -0.158 | -0.0290 | No |
| 67 | MT3 |  |  | 8329 | -0.265 | -0.0665 | No |
| 68 | S100B |  |  | 8562 | -0.373 | -0.0688 | No |
| 69 | DBI |  |  | 8571 | -0.383 | -0.0448 | No |
| 70 | RHOC |  |  | 8675 | -0.484 | -0.0250 | No |
| 71 | RAB31 |  |  | 8696 | -0.514 | 0.0063 | No |
Table: GSEA details [plain text format]

  

Fig 2: RICHARDS\_ZHONG\_NATURE\_2018\_OPC\_UPREG: Random ES distribution      
 Gene set null distribution of ES for **RICHARDS\_ZHONG\_NATURE\_2018\_OPC\_UPREG**
